# Contemporary Chinese dietary pattern: Where are the hidden risks?

**DOI:** 10.3389/fnut.2022.997773

**Published:** 2022-09-23

**Authors:** Hong Xiang, Xufeng Tao, Xi Guan, Tianyi Yin, Junchen Li, Deshi Dong, Dong Shang

**Affiliations:** ^1^Laboratory of Integrative Medicine, The First Affiliated Hospital of Dalian Medical University, Dalian, China; ^2^Department of Pharmacy, The First Affiliated Hospital of Dalian Medical University, Dalian, China; ^3^Department of General Surgery, The First Affiliated Hospital of Dalian Medical University, Dalian, China

**Keywords:** dietary risk, China, death, disability-adjusted life years, summary exposure value, Global Burden of Disease Study

## Abstract

**Background:**

With the rapid improvement in economy and lifestyle, dietary risk-related diseases have become a public health problem worldwide. However, the health effects of dietary risk over time have not been fully clarified in China. Here, we explored the temporal trends in the death burden of unhealthy dietary habits in China and benchmark dietary risk challenges in China to G20 member states.

**Method:**

Sex–age-specific burdens due to dietary risk in China were extracted from the Global Burden of Disease (GBD) Study 2019, including annual numbers and age-standardized rates (ASRs) of death, disability-adjusted life years (DALYs), and summary exposure values (SEVs) during 1990–2019. The variation trend of ASRs was evaluated by estimated annual percentage changes (EAPCs).

**Result:**

Between 1990 and 2019, the number of dietary risk-based death and DALYs increased significantly in China with an overall downward trend of ASDR and ASR-DALYs. Ischemic heart disease was the first cause of death from diet, followed by stroke and colon and rectum cancers. Chinese men were at greater risk than women for diet-related death and DALYs. Further analysis showed that a high sodium diet has always been the “No. 1 killer” that threatens the health of Chinese residents. The death burden of dietary risk demonstrated an increasing trend with age, and the peak was reached in people over 75 years. Compared with other G20 countries, Japan and South Korea have the most similar dietary patterns to China with the character of high sodium intake. Notably, decreased whole grain intake, as the primary dietary risk attributable to death and DALYs burden in the United States and European countries, had already ranked second in China's dietary risks.

**Conclusion:**

China's dietary burden cannot be ignored. Chinese residents should pay more attention to the collocation of dietary nutrients, especially men and 75+ years (elderly) people. Targeted dietary adjustments can significantly reduce deaths and DALYs in China.

## Introduction

Globally, in 2019, diet was the second leading risk attributed to deaths in women, and the third leading risk of deaths in men (1). Dietary patterns have been confirmed to be correlated with a variety of non-communicable diseases (NCDs), including cancers, cardiovascular disease, metabolic disease, etc. (2–4). A prospective cohort study reported that the intake of ultra-processed foods is positively associated with the risk of cardiovascular diseases (5). High consumption of preserved foods is linked to increased nasopharyngeal carcinoma risk in adolescence and adulthood (6). In addition, a dietary pattern with high consumption of plant foods but a minimal consumption of animal products has been recommended for dietary prevention and management of type 2 diabetes and related micro- and macrovascular complications (7).

With the rapid development in the economy and lifestyle, Chinese residents are presently undergoing unprecedented diversifications in their dietary patterns, and the focus is on high sugar and saturated fats intake (8). In order to prevent and manage diet-related diseases, a monitoring system for nutrition and risk factors covering the whole life course has been established in China, and the correlation between dietary patterns and diseases has been reported successively (9–12). However, there are still a lack of systematic national data covering the health impacts of dietary transitions and their trend variations as far as we know. In this report, we retrieve data from the Global Burden of Diseases Study (GBD) 2019 to examine the sex-age-specific burden of dietary risks-related death in China from 1990 to 2019, and benchmark dietary risk challenges in China to G20 member countries. In short, we found that the numbers of death and disability-adjusted life years (DALYs) at dietary risk increased significantly in China, while overall age-standardized rates (ASRs) of death (ASDR) and ASR-DALYs were on the decline from 1990 to 2019. The continued risk of a diet high in sodium and the dietary risk caused by the introduction of western dietary culture need more attention. In terms of individual differences, we found that male dietary risks were more serious than female ones, and age was positively correlated with most dietary risks. Therefore, this systematic investigation of unhealthy dietary habits may provide critical guidance for dietary consumption patterns to prevent and manage dietary risk-related diseases.

## Methods

### Data sources

Data on annual numbers and age-standardized rates (ASRs) of sex-age-specific death and DALYs due to dietary risk from 1990 to 2019 were collected *via* the GBD 2019 study using GBD Results Tool (https://ghdx.healthdata.org/gbd-results-tool). The GBD 2019 under the leadership of the Institute for Health Metrics and Evaluation (IHME) provides an open access tool to quantify the health burden from 369 diseases and injuries and 87 risk factors obtained from 204 countries and territories. Data coverage and statistical modeling methods for the GBD 2019 have been published in previous research (13).

### Dietary risk factor definitions

A dietary risk factor is a behavior factor causally linked to an increased or decreased probability of getting a disease or injury. The decreased probability means the risk is a protective factor. In GBD 2019, dietary risks are classified as a secondary category under behavioral risks, with a total of 15 risks as follows: low fruits (≤ 200–300 g/day), vegetables (≤ 290–430 g/day), legumes (≤ 50–70 g/day), whole grains (≤ 100–150 g/day), nuts and seeds (≤ 16–25 g/day), milk (≤ 350–520 g/day), fiber (≤ 19–28 g/day), seafood omega-3 fatty acids (≤ 200–300 g/day), polyunsaturated fatty acids (≤ 9–13% of total daily energy), and calcium (≤ 1.00–1.50 g/day) intake; and high red meat (≥18–27 g/day), processed meat (≥ 0–4 g/day), sugar-sweetened beverages (≥ 50 kCal per 226·8 serving), trans fatty acids (≥ 0.0–1.0% of total daily energy), and sodium (≥ 1–5 g/day) consumption. The exposure definition and optimal level (or range) of intake have been reported in detail elsewhere (14). The inclusion of risk factors is mainly according to the association strength among dietary risks and diseases, evidence levels, and data availability.

### Statistical analyses

All measures were reported as numbers and ASRs. An estimate of disability-adjusted life years (DALYs) is computed by summarizing the number of healthy years lost to disease, weighted for severity by disability weights (YLDs), and dividing that amount by the number of healthy years lost to the disease. Summary exposure value (SEV) measures the risk factor exposure of a population. SEV = 0, there is no excess risk for the population; and SEV = 1, the population is at the highest level of risk. According to GBD 2019, SEV is reported as a risk-weighted prevalence ranging from 0 to 100%. SEV decreases with decreasing exposure to a particular risk factor, while SEV increases with increasing exposure. Data were presented as values with a 95% uncertainty interval (UI). Moreover, we artificially divided these populations into four age groups: 25–44 years (young), 45–59 years (middle-aged), 60–74 years (middle-old aged), and 75+ years (old aged), and summed their data as the new age group (15, 16). In addition, to reflect the change trends of ASDRs from 1990 to 2019, we also calculated the estimated annual percentage change (EAPC) of ASDRs based on the formula 100 × (exp(β) – 1) (*Y* = α + β*X* + ε, *Y* = ln (ASR), *X* = calendar year, ε = the error term), and the 95% confidence interval (CI) was obtained from the linear regression model. All data were analyzed by using the R program (Version 4.2.0) or GraphPad Prism (Version 6).

## Results

### Overall effects of dietary risks on health in China

**Figure 1:**
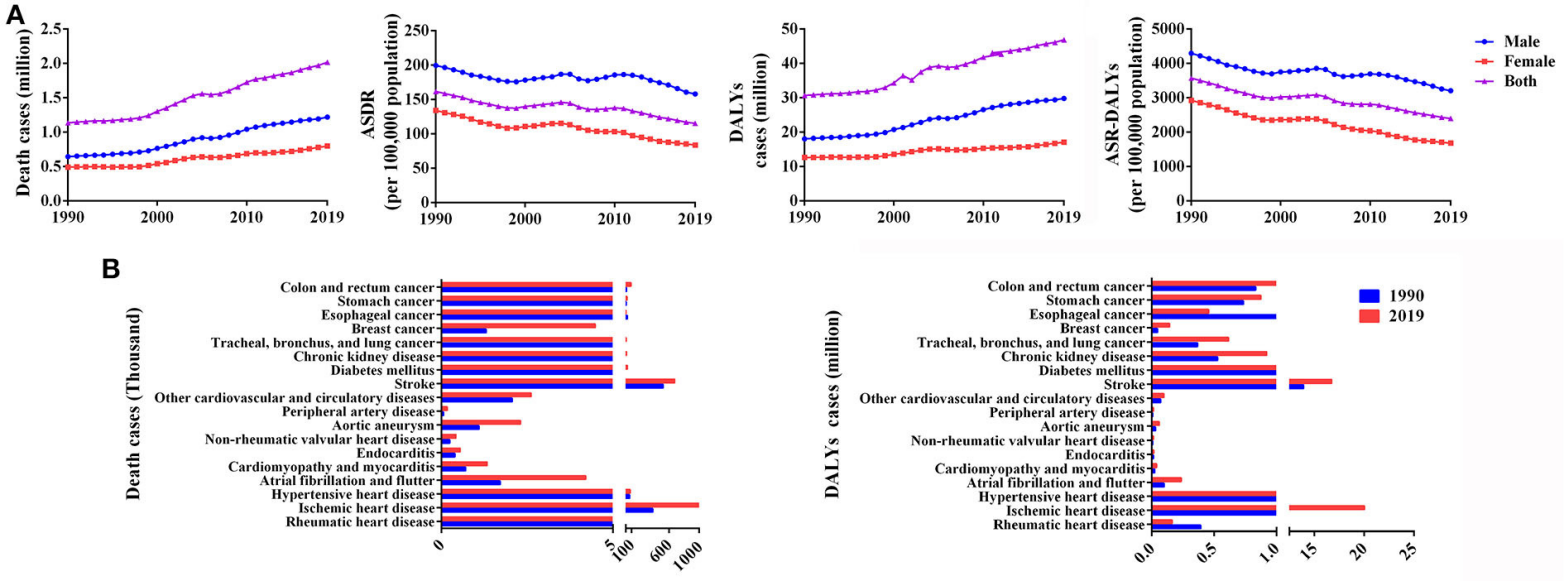
Overall effects of dietary risks on health in China from 1990 to 2019. **(A)** Deaths, ASDR, DALYs, and ASR-DALYs by sex from 1990 to 2019. **(B)** Deaths and DALYs in individual diseases in 1990 and 2019.

The effects of dietary risks on health in China were presented as the numbers of death and DALYs, and these indexes all significantly increased from 1990 to 2019 (Table 1; Supplementary Table 1). In 2019, diet-related deaths accounted for 2.015 million deaths (95% UI 1.494–2.645), with an age-standardized death rate (ASDR) of 115.054 per 100,000 (95% UI 84.966–151.654) and an EAPC of −0.874 (−1.018– −0.730) (Table 1 and Figure 1A). Notably, dietary risk-related deaths increased dramatically in men in the last three decades, reaching 1.218 million (95% UI: 0.885–1.631) in 2019, which was 1.5 times that of women (Figure 1A). Moreover, as shown in Figure 1A and Supplementary Table 1, the loss of dietary factor-related DALYs reached 46.813 million (95% UI 35.645–60.012) person-years, and the ASR-DALYs of dietary risks was 2,393.995 per 100,000 (95% UI: 1,823.458–3,070.583) in 2019. The health burden in men was more severe than in women, with ASDR and ASR-DALYs of 157.902/100,000 and 3,203.180/100,000, respectively. In addition, the EAPC of dietary risks related ASR-DALYs were −1.254 (−1.285 – −1.223), −0.711 (−0.825 – −0.597), and −1.807 (−1.930 – −1.685) in both, men and women, respectively. Ischemic heart disease was the first cause of diet-related deaths [0.997 (0.766–1.227) million deaths] and DALYs [20.044 (15.603–24.457) million DALYs], followed by stroke [0.672 (0.436–0.937) million deaths and 16.729 (11.517–22.375) million DALYs] and colon and rectum cancer [0.090 (0.066–0.115) million deaths and 2.234 (1.610–2.831) million DALYs] (Figure 1B, Table 1, and Supplementary Table 1).

**Table 1:** The death numbers and age-standardized death rates of dietary risks in China.

**Characteristics**	**1990**		**2019**		**1990–2019**
	**Number of deaths (95% UI)**	**ASDR per 100,000 (95% UI)**	**Number of deaths (95% UI)**	**ASDR per 100,000 (95% UI)**	**EAPC of ASDR**
China	1,134,883.502 (856,372.638–1,466,841.344)	161.523 (120.976–211.032)	2,015,169.973 (1,494,173.501–2,645,141.246)	115.054 (84.966–151.654)	−0.874 (−1.018– −0.730)
**Sex**
**Men**	643,240.967 (478,196.989–837,808.740)	199.3180424 (148.896–259.249)	1,217,584.924 (884,975.215–1,631,263.425)	157.902 (116.2721–209.978)	−0.404 (−0.556– −0.251)
Women	491,642.535 (359,891.326–658,708.149)	133.796 (98.314–180.775)	797,585.049 (554,673.6788–1,110,938.342)	83.689 (58.012–116.222)	−1.382 (−1.547– −1.216)
Dietary risk-related diseases
Rheumatic heart disease	12,491.670 (5,015.431–24,615.362)	1.497 (0.569–3.035)	5,529.203 (1,948.707–11,472.504)	0.287 (0.097–0.602)	−5.513 (−5.632– −5.394)
Ischemic heart disease	381,013.400 (310,915.419–448,758.930)	58.806 (47.864–70.269)	997,011.686 (766,215.915–1,227,448.978)	60.037 (46.276–74.239)	0.693 (0.430–0.957)
Hypertensive heart disease	71,590.602 (24,382.420–154,000.354)	10.830 (2.934–24.805)	77,818.671 (19,528.343–187,735.101)	4.582 (0.996–11.787)	−2.919 (−3.736– −2.095)
Atrial fibrillation and flutter	1,710.756 (593.410–3,226.251)	0.320 (0.092–0.645)	4,209.944 (1,250.690–8,718.238)	0.268 (0.069–0.579)	−0.657 (−0.773– −0.540)
Cardiomyopathy and myocarditis	705.917 (271.383–1,473.184)	0.081 (0.029–0.177)	1,331.259 (548.031–2,456.932)	0.069 (0.027–0.128)	−0.232 (−0.520–0.058)
Endocarditis	391.182 (175.613–688.914)	0.044 (0.019–0.080)	545.547 (234.071–985.726)	0.028 (0.012–0.051)	−2.141 (−2.415– −1.867)
Non-rheumatic valvular heart disease	239.849 (95.331–434.226)	0.026 (0.010–0.048)	421.215 (185.761–732.455)	0.020 (0.009–0.036)	−0.967 (−1.030– −0.904)
Aortic aneurysm	1,096.117 (467.719–1,966.616)	0.130 (0.053–0.239)	2,306.974 (983.398–4,095.032)	0.114 (0.048–0.207)	−0.419 (−0.493– −0.345)
Peripheral artery disease	54.730 (20.018–103.782)	0.008 (0.003–0.017)	169.980 (59.887–322.774)	0.009 (0.003–0.019)	0.504 (0.379–0.630)
Other cardiovascular and circulatory diseases	2,062.254 (924.023–3,619.849)	0.257 (0.107–0.477)	2,618.109 (1,056.149–4,654.357)	0.136 (0.051–0.248)	−2.161 (−2.287– −2.035)
Stroke	519,444.502 (365,946.892–684,456.414)	71.307 (49.378–95.394)	671,872.079 (436,354.759–937,093.269)	36.313 (23.549–50.850)	−2.311 (−2.478– −2.144)
Diabetes mellitus	16,985.751 (13,217.478–21,229.306)	2.282 (1.789–2.853)	41,951.856 (31,431.378–52,994.938)	2.269 (1.702–2.847)	0.121 (−0.118–0.360)
Chronic kidney disease	14,911.092 (6,753.376–25,100.603)	1.903 (0.800–3.346)	29,665.725 (11,330.585–54,546.973)	1.564 (0.557–2.956)	−0.312 (−0.455– −0.168)
Tracheal, bronchus, and lung cancer	13,486.463 (4,581.842–20,861.271)	1.640 (0.555–2.535)	27,187.203 (7,159.651–43,057.971)	1.402 (0.368–2.221)	−0.464 (−0.720– −0.207)
Breast cancer	1,302.360 (455.731–1,907.567)	0.144 (0.049–0.210)	4,483.464 (2,013.556–6,484.881)	0.223 (0.098–0.322)	1.672 (1.594–1.751)
Esophageal cancer	40,514.343 (17,910.852–66,618.102)	5.066 (2.230–8.319)	20,508.962 (4,338.342–52,320.932)	1.074 (0.237–2.733)	−5.744 (−6.224– −5.261)
Stomach cancer	27,226.500 (612.935–101,649.434)	3.343 (0.077–12.562)	37,131.477 (833.143–138,478.719)	1.900 (0.043–7.120)	−1.718 (−2.108– −1.328)
Colon and rectum cancer	29,656.014 (23,146.429–35,344.014)	3.839 (3.015–4.567)	90,406.618 (65,690.670–114,669.415)	4.761 (3.475–6.008)	1.112 (0.851–1.374)

### SEV and risk-specific trends of individual dietary risk factors

Age-standardized SEV rate of dietary risks declined in China (ARC = −0.055, 95% UI: −0.099– −0.019), falling from 81.860 per 100,000 people in 1990 (95% UI: 75.436–85.149) to 77.367 per 100,000 people in 2019 (95% UI: 69.577–81.933). Overall, men had a lower rate of change (ARC = −0.048, 95% UI: −0.089– −0.010) than women (ARC = −0.062, 95% UI: −0.122– −0.013). Based on the analysis of different individual risk factors of diet, the decreased age-standardized SEV rates of high trans fatty acids, sodium, and sugar-sweetened beverages consumption, as well as a diet low in calcium, legumes, fruits, nuts and seeds, fiber, seafood omega-3 fatty acids and polyunsaturated fatty acids in 2019 compared with those in 1990 have been reported, in particular, the biggest reduction was in the intake of low-vegetable diets. Conversely, the age-standardized SEV rates for four dietary risks in 2019 were higher than those in 1990, including a diet high in red meat and processed meat, but low in whole grains and milk. The details are integrated into Table 2.

**Table 2:** The age-standardized SEV rates of dietary risks in 1990 and 2019, and annualized rate of changes in China.

**Factors**	**Sex**	**Age-standardized SEV rate (95% UI)**		**ARC (%) (95% UI)**
		**1990**	**2019**	
Dietary risks	Male	83.875 (77.838–87.072)	79.849 (72.971–84.005)	−0.048 (−0.089– −0.010)
	Female	79.976 (72.903–84.025)	74.997 (65.946–80.617)	−0.062 (−0.122– −0.013)
	Both	81.860 (75.436–85.149)	77.367 (69.577–81.933)	−0.055 (−0.099– −0.019)
Diet high in trans fatty acids	Male	42.712 (34.609–58.711)	36.310 (28.414–52.930)	−0.150 (−0.267– −0.039)
	Female	43.229 (35.114–59.625)	37.092 (29.163–55.484)	−0.142 (−0.262– −0.037)
	Both	42.968 (35.388–58.614)	36.703 (29.400–53.605)	−0.146 (−0.239– −0.053)
Diet high in sodium	Male	96.270 (89.258–98.506)	95.227 (87.092–98.274)	−0.011 (−0.052–0.018)
	Female	91.123 (82.062–95.811)	88.888 (77.651–94.938)	−0.025 (−0.097–0.034)
	Both	93.570 (85.894–96.741)	91.959 (82.299–96.237)	−0.017 (−0.063–0.016)
Diet high in red meat	Male	40.009 (28.462–51.287)	70.206 (60.815–78.513)	0.755 (0.437–1.299)
	Female	40.771 (29.598–51.974)	71.187 (60.778–79.408)	0.746 (0.445–1.311)
	Both	40.381 (29.656–50.881)	70.696 (61.870–78.158)	0.751 (0.472–1.225)
Diet high in processed meat	Male	8.246 (3.869–18.999)	15.160 (6.528–32.013)	0.838 (0.169–1.513)
	Female	8.940 (4.043–19.284)	17.893 (8.077–35.478)	1.002 (0.317–1.775)
	Both	8.585 (3.978–19.220)	16.516 (7.344–33.916)	0.924 (0.211–1.577)
Diet high in sugar-sweetened beverages	Male	34.798 (23.186–56.595)	25.477 (17.310–40.130)	−0.268 (−0.479– −0.031)
	Female	30.751 (20.024–50.824)	23.801 (15.697–37.188)	−0.226 (−0.488–0.092)
	Both	32.804 (22.192–53.784)	24.609 (16.831–38.618)	−0.250 (−0.472– −0.020)
Diet low in calcium	Male	71.102 (59.686–85.775)	48.105 (34.432–67.585)	−0.323 (−0.444– −0.167)
	Female	65.386 (53.300–81.907)	44.725 (31.127–64.809)	−0.316 (−0.435– −0.161)
	Both	68.279 (56.874–83.824)	46.380 (32.989–65.969)	−0.321 (−0.439– −0.164)
Diet low in legumes	Male	68.592 (27.649–96.869)	56.836 (15.073–87.372)	−0.171 (−0.520– −0.042)
	Female	64.949 (23.103–94.802)	50.112 (11.598–79.793)	−0.228 (−0.563– −0.065)
	Both	66.802 (25.641–95.586)	53.474 (13.665–82.957)	−0.200 (−0.529– −0.062)
Diet low in fruits	Male	73.895 (65.310–83.149)	46.175 (35.627–58.284)	−0.375 (−0.492– −0.267)
	Female	74.088 (66.107–82.950)	44.341 (33.928–56.542)	−0.402 (−0.517– −0.293)
	Both	74.004 (66.377–82.688)	45.261 (34.847–57.180)	−0.388 (−0.496– −0.282)
Diet low in vegetables	Male	40.503 (23.951–59.575)	3.467 (1.790–9.987)	−0.914 (−0.954– −0.759)
	Female	39.486 (22.936–58.669)	3.226 (1.666–9.468)	−0.918 (−0.957– −0.757)
	Both	39.993 (23.508–58.867)	3.343 (1.759–9.544)	−0.916 (−0.955– −0.751)
Diet low in whole grains	Male	82.563 (75.203–90.092)	84.068 (77.218–91.031)	0.018 (0.005–0.036)
	Female	80.516 (72.192–88.364)	82.163 (74.147–89.815)	0.020 (0.005–0.044)
	Both	81.552 (73.652–89.237)	83.111 (75.692–90.380)	0.019 (0.008–0.035)
Diet low in nuts and seeds	Male	64.907 (32.562–81.146)	43.074 (18.872–65.409)	−0.336 (−0.560– −0.160)
	Female	67.234 (33.953–82.902)	45.690 (20.034–67.532)	−0.320 (−0.548– −0.159)
	Both	66.048 (33.217–81.898)	44.382 (18.625–66.423)	−0.328 (−0.543– −0.165)
Diet low in milk	Male	94.508 (88.302–99.043)	96.002 (88.915–100.000)	0.016 (−0.012–0.048)
	Female	94.639 (88.337–99.270)	95.651 (88.066–100.000)	0.011 (−0.015–0.039)
	Both	94.569 (88.259–99.154)	95.827 (88.545–100.000)	0.013 (−0.013–0.044)
Diet low in fiber	Male	37.193 (24.425–50.594)	19.658 (10.693–29.732)	−0.471 (−0.620– −0.311)
	Female	40.044 (25.811–53.939)	22.406 (12.763–33.065)	−0.440 (−0.596– −0.276)
	Both	38.611 (25.529–51.752)	21.021 (12.611–30.549)	−0.456 (−0.580– −0.332)
Diet low in seafood omega-3 fatty acids	Male	99.745 (99.314–99.996)	96.338 (91.803–99.959)	−0.034 (−0.078– −0.000)
	Female	99.813 (99.510–99.998)	97.162 (93.146–99.990)	−0.027 (−0.065–0.000)
	Both	99.778 (99.468–99.996)	96.747 (92.791–99.974)	−0.030 (−0.068– −0.000)
Diet low in polyunsaturated fatty acids	Male	87.430 (69.042–95.867)	67.899 (36.442–91.612)	−0.223 (−0.483–0.024)
	Female	88.299 (71.746–95.898)	69.446 (38.411–91.784)	−0.214 (−0.464–0.018)
	Both	87.859 (70.390–95.926)	68.683 (37.169–91.776)	−0.218 (−0.467–0.019)

### Impact of individual factors of dietary risk on death burden

**Figure 2:**
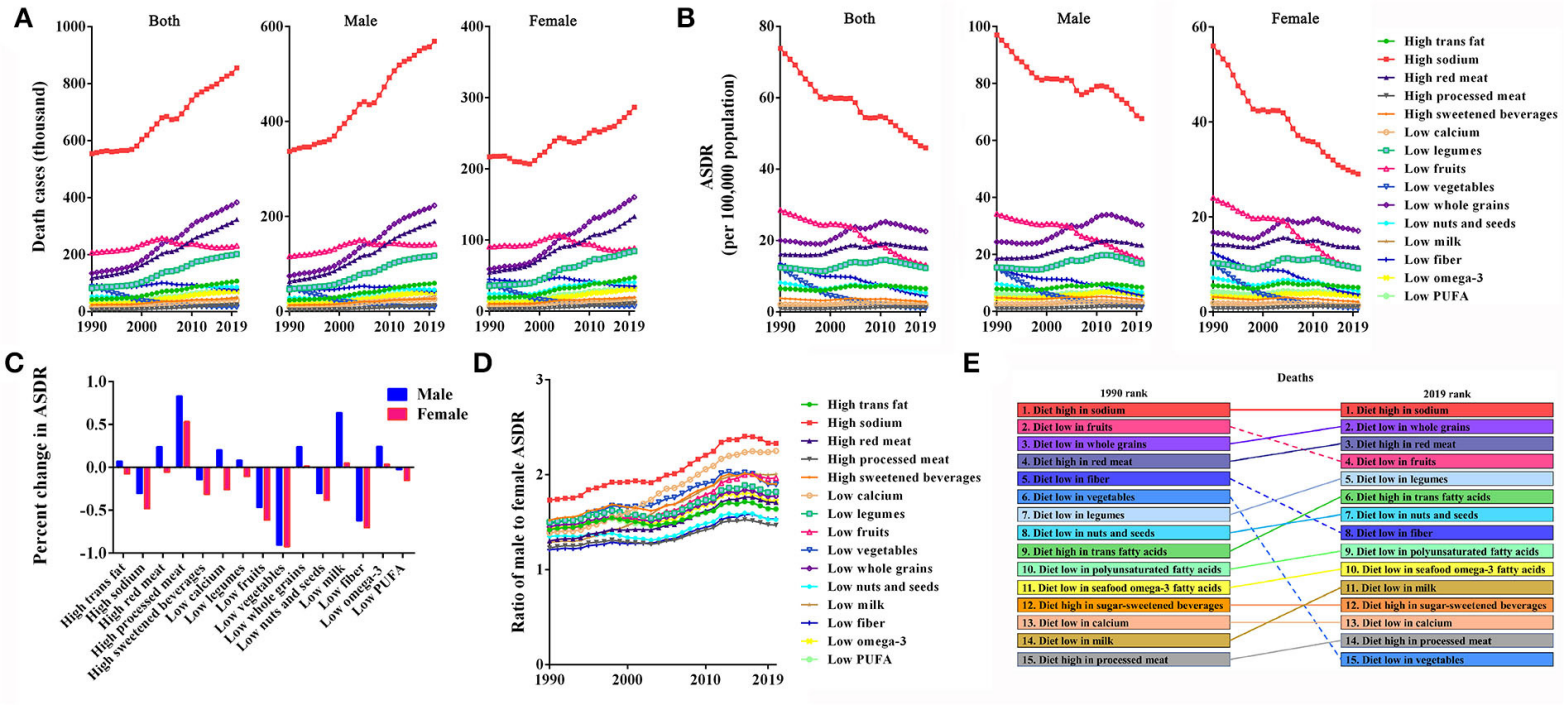
Impact of individual factors of dietary risk on death burden in China from 1990 to 2019. **(A,B)** Deaths and ASDRs in individual factors of dietary risk by sex. **(C)** Percent changes in ASDRs in men and women. **(D)** Male-to-female ratios of ASDRs in individual factors of dietary risk. **(E)** Ranking of deaths due to individual dietary risk factors.

From 1990 to 2019, the impact of different dietary risks on the death burden varied widely. As shown in Figure 2A, deaths from a diet with high sodium and red meat, and low whole grains and legumes continued to climb, and the remaining dietary risk-related deaths have gradually plateaued, including high consumption of trans fatty acids, sugar-sweetened beverages, and diet with low calcium, fruits, nuts and seeds, milk, seafood omega-3 fatty acids and polyunsaturated fatty acids, etc. In China, high sodium intake-related deaths have long topped the list. In 1990, 0.554 million Chinese died from a high-sodium diet, but 0.855 million died in 2019. A high-sodium diet killed far more Chinese men than women.

Contrary to the rise in death cases, with the increase of the Chinese population, the majority of ASDR of dietary risks showed an overall volatile downward trend, 1990–2019, in which, the death burden due to a high sodium diet, and diets low in fruits, fiber and vegetables were significantly reduced (Figure 2B). There are gender differences in ASDRs of dietary risks in China, and the typical feature was that the ASDRs of high sodium, low fruit, fiber, and vegetable intake in Chinese men were higher than that of women (Figure 2B). Additionally, the ASDR percent change in women over the past 30 years decreased more significantly than in men (Figure 2C). From 1990 to 2019, the ratios of male ASDR to female ASDR were essentially maintained between 1.2 and 1.8 across 15 dietary risk factors. However, the ratio of sodium-rich and calcium-deficient diets reached over 2.0 (Figure 2D), indicating a further disparity between sexes in death burden due to high sodium and low calcium consumption. It is a relatively serious problem that the burden of dietary risks on health appears to be higher in men than in women.

As shown in Figure 2E, the dietary structure and eating habits of Chinese residents have undergone great changes over the study period with the improvement of living standards. The most significant improvement was the rank of death burden due to low fruits, fiber, and vegetable intake dropping from 2nd, 5th, and 6th in 1990 to 4th, 8th, and 15th in 2019, respectively. Under the influence of western animal-source diets, the death burden of Chinese residents due to a diet high in red meat has risen from the 4th in 1990 to the 3rd in 2019, and the ranking of other dietary risk factors all increased by varying degrees. From 1990 to 2019, the deaths due to a diet high in sodium have always ranked first, and it has become the “No. 1 killer” that threatens the health of Chinese residents.

### Burden of dietary risk-related deaths in various age groups

**Figure 3:**
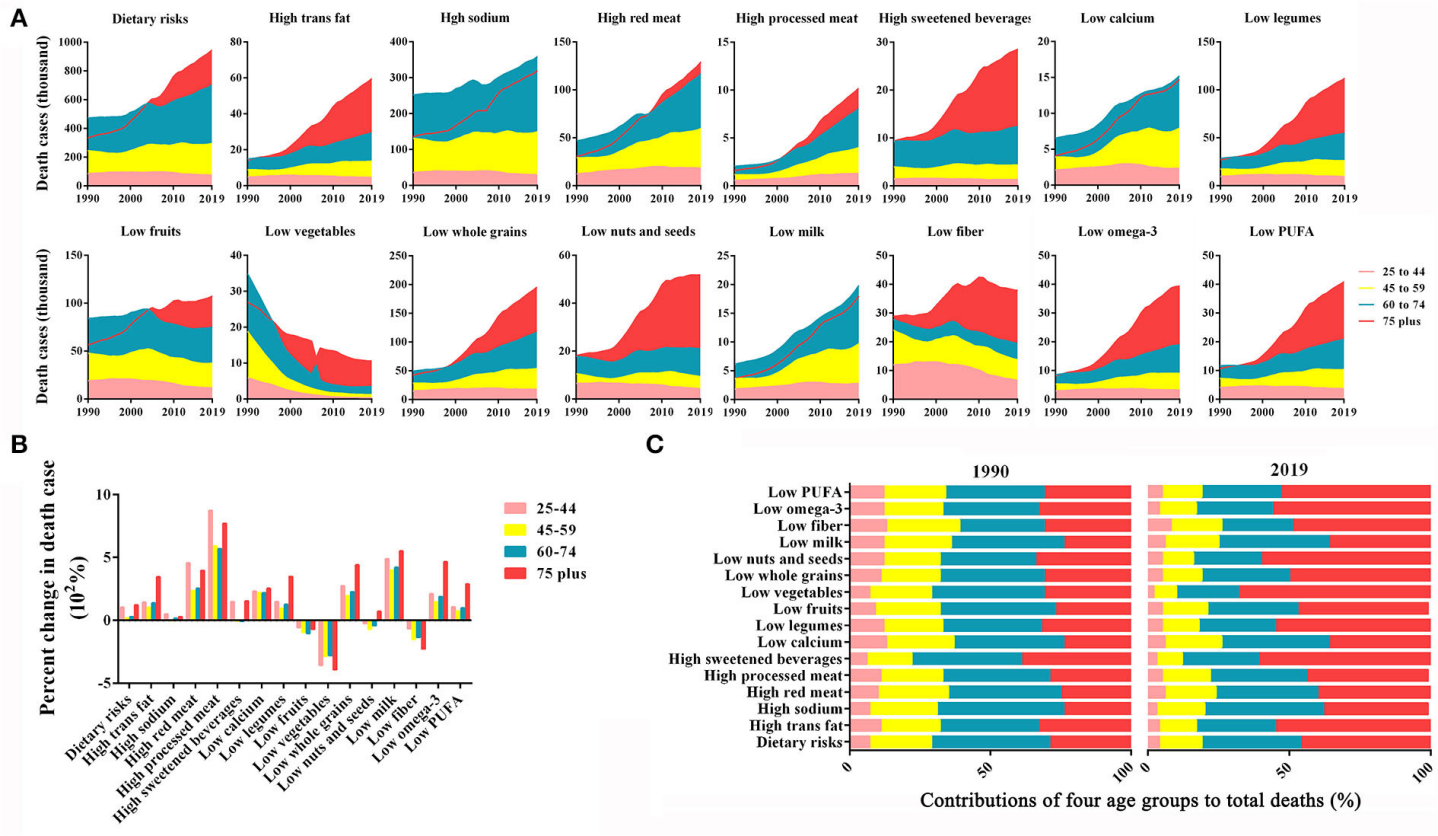
Burden of dietary risk-related deaths in various age groups of China in individual dietary risk factors from 1990 to 2019. **(A)** The contribution of each age group to total deaths. **(B)** The percent changes in deaths in the four age groups. **(C)** The four age groups as percentages of total deaths.

As shown in Figure 3A, the Chinese death burden of dietary risks demonstrated age-related increases. Most dietary risk-related deaths occurred in the senior population, particularly those ≥ 75 years of age (old age). It was the old age group that had the highest absolute number of deaths in 2019 due to diets high in trans fatty acids, red meat, processed meat, sugar-sweetened beverages, and diets low in legumes, fruits, vegetables, whole grains, nuts, and seeds, fiber, seafood omega-3 fatty acids, polyunsaturated fatty acids, respectively; and the middle-old aged group had the highest absolute levels of deaths due to a diet high in sodium, and a diet low in calcium and milk. Comparatively to the other dietary risk factors, a sodium-rich diet led to the highest number of deaths at the end of the study period.

Next, the corresponding percent changes in death cases of each group for each dietary risk factor during 1990–2019 were examined and contrasted. Over the study period, the old-aged group in China experienced the greatest increase in deaths, followed by the young group (Figure 3B). All age groups showed marked increases in deaths due to a diet high in processed meat, while a diet low in fruits, vegetables, and fiber demonstrated a full-scale decline.

A trend toward older ages was observed in deaths due to dietary risks between 1990 and 2019. The middle-old aged group had the highest proportion of dietary risk-related deaths in 1990 until it was fully overtaken by the elderly individuals in 2019, with the contributions of the youth and middle-aged groups to total deaths due to decreased dietary risk (Figure 3C). A diet low in fiber had the youngest distribution among all dietary risk factors, as a fiber-deficiency diet in young and middle-aged groups accounted for as many as 39% of deaths in 1990 and 26% in 2019. In contrast, the distribution of diets poor in vegetables was the oldest, with middle-aged and elderly people accounting for 71% of deaths in 1990 and 90% in 2019.

### Comparative view of dietary risk-related death burden across the G20 states

**Figure 4:**
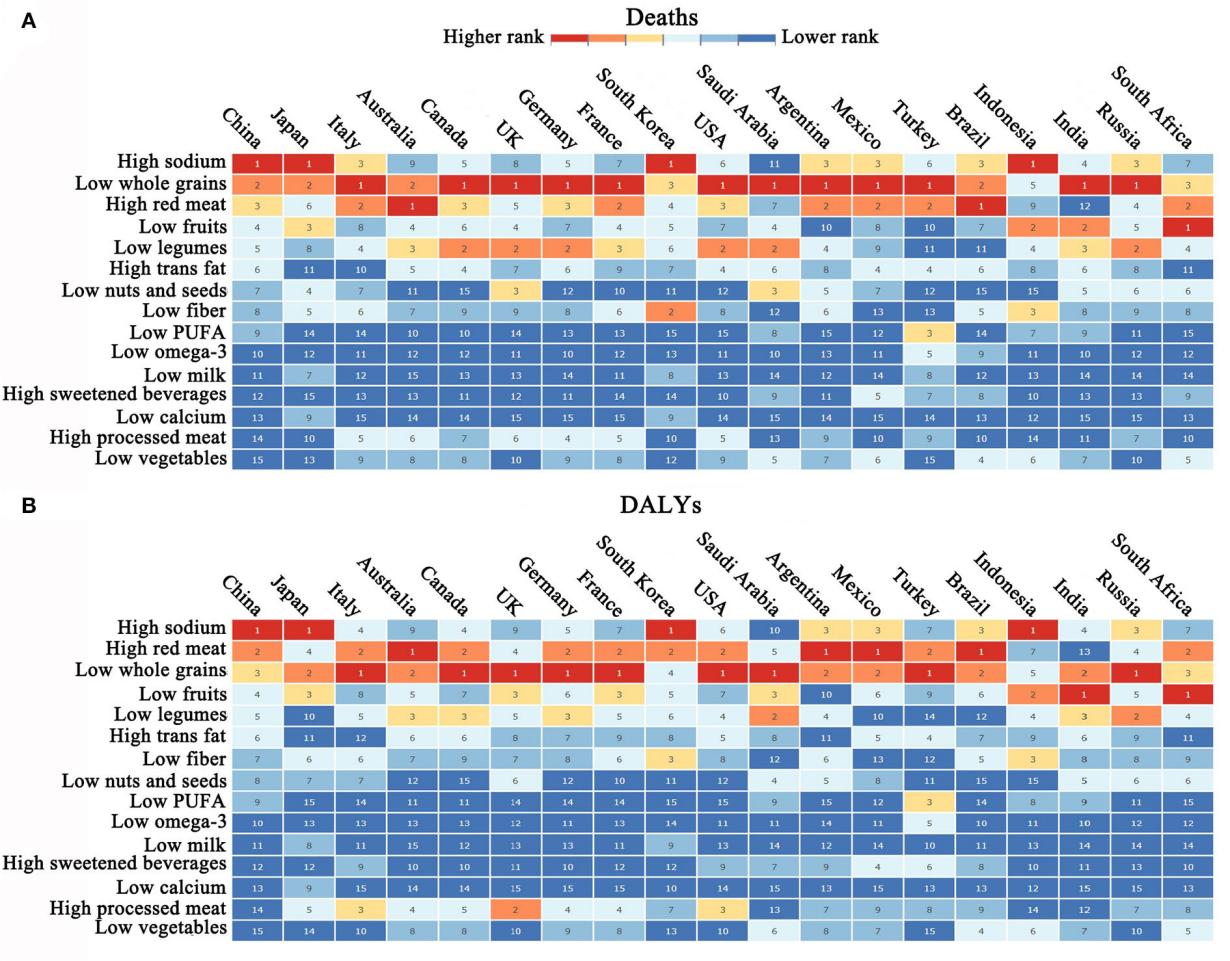
Comparative view of dietary risk-related death burden across the G20 states [This figure was sourced from GBD 2019 study (https://vizhub.healthdata.org/gbd-compare/)]. **(A,B)** Ranking of dietary risk-related death and DALYs in the G20 states.

A comparison of the death burden caused by dietary risk in China and other G20 members is shown in Figures 4A,B. In China, the top three deaths and DALYs caused by dietary risk factors were due to eating too much sodium, eating few whole grains, and consuming too much processed meat. Compared with the G20 countries, Japan and Korea have the most similar dietary patterns to China. Traditional diets in East and South-East Asia have an obvious characteristic with dying high in sodium; therefore, high sodium intake-related dietary risks have always been associated with the highest deaths and DALYs in China, Japan, South Korea, and Indonesia. However, a diet low in whole grains is the major dietary risk associated with death, and DALYs in the United States and European countries had ranked second in China, only behind a diet high in sodium, suggesting that Chinese traditional eating habits are facing challenges from western diets.

## Discussion

Diet-related disease burden has been widely documented, but not well-understood in China. Data on dietary risk factors related to health burden in China were comprehensively reviewed in this study, and the trend of the health impacts of dietary risk was presented annually by the numbers and ASRs of death and DALYs. From 1990 to 2019, dietary risk factors had become major challenges that seriously threaten the health of Chinese residents. The number of death and DALYs due to dietary risk increased significantly in China, but the ASDR and ASR-DALYs showed overall downward trends with the proportion growth of the elderly population. It is vigilant that dietary intakes differ by gender contributing to differences in death burden between men and women, and the gap is widening year by year. Compared with Chinese women, men have a heavier dietary risk-related burden as a result of unhealthy eating. Women have been reported a better diet quality than men, with better eating behavior and healthier weight management, which can be reflected in lower energy density, higher carbohydrate energy ratio, and higher frequency of cooking in women's diet. Conversely, the established impression is that men consume more lipids and fewer carbohydrates than women (17, 18). Moreover, compared with women, men reported less favorable attitudes and a weaker sense of behavioral control regarding fruit and vegetable intake, let alone perception of the importance of health (19). It is likely that the gap between the sexes will continue to widen in the future if this trend continues. Thus, the significant gender difference in dietary risk-related death should be taken into account in national prevention programs.

In the past 3 decades, the influence of traditional Chinese food culture on Chinese dietary structure is still strong. It is encouraging to see the age-standardized SEV rate for diets high in sodium gradually decreasing, but the sodium intake is still twice the World Health Organization's (WHO) tolerable upper intake (<2.0 g/d), and home food preparation accounted for more than 67% of sodium intake (20). Sodium-rich diet was also responsible for the majority of diet-related deaths, which is similar to that of Japan and Korea which are members of the G20 along with China. According to data from the WHO, the consumption of high sodium levels has been linked to a number of NCDs in adults and children, such as cardiovascular disease, hypertension, and stroke, and the reduction of sodium intake is a cost-effective public health intervention for preventing NCDs (21). In China, ischemic heart disease was the chief cause of diet-related death, followed by stroke. According to a meta-analysis of 616,905 participants, dietary sodium intake and cardiovascular disease risk are significantly correlated, and an increase in sodium intake of 1 g was associated with an increase in cardiovascular disease risk of up to 6% (22). Health outcomes and healthcare costs can be significantly improved by reducing dietary salt by 3 g per day, according to the Coronary Heart Disease Policy Model (23). Therefore, a low-sodium diet should be a public health goal of the Chinese Health Sector, and education on sodium-reduced intake should be promoted. In addition, the dietary structure of Chinese residents is facing the challenges of western eating habits, mainly manifested in the rising number of deaths from high red meat and processed meat consumption but low whole grains intake during 1990–2019. In prospective studies, consumption of red and processed meat is associated with an increased risk of ischemic heart disease, and such effects could be mediated by the association between red and processed meat and plasma non-high-density lipoprotein cholesterol and systolic blood pressure. Every 100 g of red or processed meat consumed each day would increase the risk of colorectal cancer by 12%, and the carcinogenic potential of these foods can be summarized as the presence of carcinogens or their precursors produced by food processing. However, the risk of colorectal cancer decreases by 17% with each 90 g/day increase in whole grains (24–26). Western diet represented by highly red meat and processed foods may promote diverse forms of NCDS through gut microbiome-mediated inflammatory responses; on the contrary, diets rich in whole grains without altering the gut microbiome significantly reduce systemic low-grade inflammation (27, 28). Thus, Chinese residents should pay attention to choosing healthy dietary habits and avoid excessive intake of unhealthy foods when they are enjoying the fresh experience brought by the fusion of China and the West. Although the challenges are still severe, the Chinese government's great efforts and achievements in improving the livelihood and health awareness of residents in the past 30 years are still worthy of recognition. The most significant improvement is reflected by the significantly decreased burden of death linked to low fruit and vegetable intake.

Here, the burden of dietary risk-related deaths in various age groups was also fully evaluated. Seniors (60–74 years and 75+ years) had the highest absolute numbers of deaths across all dietary risk factors at the end of the study period, and the greatest increase in deaths occurred in 75+ years within the study period. This is an inevitable result of the accelerated aging of China's population and the sharp rise in the proportion of old age. Therefore, policymakers should place dietary interventions at the top of their priority list for the foreseeable future. A diet low in fruits and vegetables demonstrated a full-scale decline; however, the problem of low vegetable intake in people over 75 years was still significant, which is because the poor chewing ability of the elderly destroys the desire for vegetable intake. Caregivers can help to reduce this death burden by learning to make more soft, chewy, and low-nutrient-dense vegetable foods for the senior population. It is vigilant that young people like to pursue sensory pleasures, pay less and less attention to diet matching, and avoid dietary risks, resulting in a significant upgrade in the percentage of deaths caused by dietary risks in the 25–44 years.

In conclusion, dietary risk continues to pose a major public health threat in China. The health effects of dietary risk caused by gender differences are serious, and the dietary risk-related death burden was much higher in men than in women, contrary to global survey data. The gap between the sexes will likely widen further if this trend continues. Sodium-rich diets are also the leading causes of diet-related deaths at the end of the study period. Furthermore, the Chinese diet is excessively influenced by western dietary culture, represented by high red and processed meat intake, which is widely reported to be associated with a variety of NCDS. Age is also an important reference for assessing the burden of diet-related deaths. The elderly were the key population for dietary interventions, followed by young adults. GBD provides comprehensive and objective data that can guide the designation of policies and measures to effectively ameliorate the negative effects of dietary risk. Considering that eating habits are very complex personal behaviors, analyzing the health effects of a certain dietary risk alone may lead to ignoring the superposition of certain factors. In the future, it is necessary to establish an evaluation model for joint analysis of multiple dietary risk factors.

## Limitations of the study

There are some limitations in this study although the GBD database provides a relatively detailed estimate of China's dietary burden. Similar to previous reports (1, 15), the data came from various regions and countries may have huge differences in the accuracy, quality, comparability, and the extent of data loss, which inevitably caused the value deviations, even though several statistical methods are used to adjust the data as much as possible. In addition, our analysis of the dietary burden in China is implemented at the national level without further analyzing local features, such as the differences between urban and rural areas.

## Data availability statement

The datasets presented in this study can be found in online repositories. The names of the repository/repositories and accession number(s) can be found in the article/Supplementary material.

## Ethics statement

Ethical review and approval was not required for the study on human participants in accordance with the local legislation and institutional requirements. Written informed consent for participation was not required for this study in accordance with the national legislation and the institutional requirements.

## Author contributions

HX and XT conceived, analyzed, drew, and drafted the manuscript. XG contributed to data analysis. TY and JL consistently contributed to references as well as drew the figures. DD edited the manuscript for submission. DS reviewed and approved the submitted manuscript. All authors contributed to the article and approved the submitted version.

## Funding

This work was financially supported by grants from the National Natural Science Foundation of China (No. 82004023), the National Key Research and Development Program of China (No. 2018YFE0195200), and the Scientific Research Project of the Department of Education of Liaoning Province (No. LZ2020075).

## Conflict of interest

The authors declare that the research was conducted in the absence of any commercial or financial relationships that could be construed as a potential conflict of interest.

## Publisher's note

All claims expressed in this article are solely those of the authors and do not necessarily represent those of their affiliated organizations, or those of the publisher, the editors and the reviewers. Any product that may be evaluated in this article, or claim that may be made by its manufacturer, is not guaranteed or endorsed by the publisher.
