# Supplementary material for: Contemporary Chinese dietary pattern: Where are the hidden risks?
Source: Front Nutr. 2022 Sep 23;9:997773. doi: 10.3389/fnut.2022.997773 (PMC9544811; doi:10.3389/fnut.2022.997773)
Supplement: Supplementary file 1 [file Table_1.DOCX]

**Table S1 The cases and age-standardized rates of disability-adjusted life years of dietary risks in China**

|  | 1990 | | 2019 | | 1990-2019 |
| --- | --- | --- | --- | --- | --- |
| Characteristics | Number of DALYs  (95% UI) | ASR-DALYs per 100,000 (95% UI) | Number of DALYs  (no. ×10^3^) (95% UI) | ASR-DALYs per 100,000 (95% UI) | EAPC of ASR-DALYs |
| China | 30634624.030  (23732943.630-38681629.430) | 3569.762  (2740.479-4544.259) | 46813125.980  (35644727.990-60011858.320) | 2393.995  (1823.458-3070.583) | -1.254  (-1.285 to -1.223) |
| Sex | | | | | |
| Males | 18012352.140  (13703318.590- 23057892.970) | 4291.476  (3274.184-5492.520) | 29750401.900  (22239936.580-39140950.040) | 3203.180  (2394.185-4203.177) | -0.711  (-0.825 to -0.597) |
| Females | 12622271.890  (9493679.987- 16434163.630) | 2924.523  (2188.016-3849.294) | 17062724.080  (12144797.040-23050304.090) | 1677.41057  (1205.242-2265.804) | -1.807  (-1.930 to -1.685) |
| Dietary risk-related diseases | | | | | |
| Rheumatic heart disease | 392496.292  (170264.489-720718.317) | 40.89591864  (17.265-78.223) | 163882.619  (68535.193-304358.192) | 8.161  (3.342-15.237) | -5.488  (-5.599 to -5.378) |
| Ischemic heart disease | 9779203.391  (7991731.552-11482434.200) | 1176.773  (965.722-1375.508) | 20043611.080  (15602998.460-24457137.300) | 1060.953  (832.243-1288.449) | 0.134  (-0.067 to 0.335) |
| Hypertensive heart disease | 1699358.727  (691276.644-3291472.649) | 210.753  (78.179-434.125) | 1635482.355  (584319.841-3454553.921) | 84.816  (27.896-187.844) | -3.111  (-3.831 to -2.386) |
| Atrial fibrillation and flutter | 97910.737  (41099.970-174381.410) | 12.460  (4.917-23.188) | 235484.586  (89136.783-428566.694) | 11.713  (4.255-21.658) | -0.149  (-0.302 to 0.005) |
| Cardiomyopathy and myocarditis | 23395.182  (9503.053-47016.724) | 2.392  (0.963-4.796) | 39194.996  (17689.722-67988.712) | 1.945  (0.869-3.379) | -0.508  (-0.751 to -0.265) |
| Endocarditis | 13231.095  (6118.706-23231.131) | 1.3140  (0.597-2.297) | 16101.121  (7426.679-27951.502) | 0.802  (0.368-1.396) | -2.246  (-2.543 to -1.948) |
| Non-rheumatic valvular heart disease | 7537.151  (3073.026-13521.442) | 0.765  (0.304-1.382) | 13110.747  (6080.039-22424.562) | 0.636  (0.293-1.089) | -0.752  (-0.858 to -0.645) |
| Aortic aneurysm | 30639.499  (13749.957-52629.443) | 3.281  (1.437-5.757) | 60017.897  (28753.719-102358.344) | 2.911  (1.366-4.976) | -0.342  (-0.434 to -0.250) |
| Peripheral artery disease | 6560.116  (2146.574-14271.808) | 0.824  (0.256-1.829) | 14558.072  (4667.172-31600.167) | 0.715  (0.224-1.565) | -0.605  (-0.641 to -0.569) |
| Other cardiovascular and circulatory diseases | 69549.919  (34609.085-115280.987) | 7.433  (3.572-12.564) | 96012.413  (45099.860-160333.426) | 4.723  (2.168-7.961) | -1.436  (-1.535 to -1.337) |
| Stroke | 13921162.200  (10116645.990-17796087.020) | 1604.943  (1154.447-2075.743) | 16729078.070  (11517379.200-22374535.750) | 833.773  (572.492-1119.879) | -2.262  (-2.382 to -2.140) |
| Diabetes mellitus | 1048787.025  (769115.036-1372981.865) | 116.875  (85.727-152.007) | 2522819.683  (1817116.691-3372078.520) | 124.591  (89.638-166.491) | 0.344  (0.143 to 0.546) |
| Chronic kidney disease | 528326.023  (264395.529-859685.813) | 57.37918093  (27.396-95.151) | 922143.088  (407985.787-1570598.137) | 45.410  (19.550-78.195) | -0.327  (-0.477 to -0.176) |
| Tracheal, bronchus, and lung cancer | 366971.697  (126354.723-566753.022) | 6.368  (6.085-6.608) | 615632.927  (162363.796-973467.258) | 7.842  (7.173-8.404) | -1.009  (-1.249 to -0.769) |
| Breast cancer | 45842.388  (16235.228-66638.218) | 2.874  (2.682-3.0609) | 143030.187  (65825.842-205621.441) | 5.028  (4.482-5.633) | 1.532  (1.476 to 1.588) |
| Esophageal cancer | 1034489.319  (449013.018-1696654.574) | 1.913  (1.621-2.180) | 455089.978  (90540.148-1163817.121) | 3.754  (3.437-4.088) | -6.147  (-6.646 to -5.646) |
| Stomach cancer | 734447.936  (16388.164-2731936.047) | 1.707  (1.355-2.044) | 873813.190  (19283.134-3220231.819) | 2.719  (2.382-3.088) | -2.004  (-2.397 to -1.610) |
| Colon and rectum cancer | 834715.335  (646748.221-998670.345) | 8.524  (8.121-8.721) | 2234062.970  (1609962.279-2831239.701) | 9.642  (8.830-10.158) | 1.061  (0.804 to 1.319) |
